# Birth and Resuscitation of (p)ppGpp Induced Antibiotic Tolerant Persister Cells

**DOI:** 10.1038/s41598-019-42403-7

**Published:** 2019-04-15

**Authors:** Mikkel Skjoldan Svenningsen, Alexandra Veress, Alexander Harms, Namiko Mitarai, Szabolcs Semsey

**Affiliations:** 1 Niels Bohr Institute, University of Copenhagen, Blegdamsvej 17, 2100 København Ø, København, Denmark; 2 Centre for Bacterial Stress Response and Persistence, Department of Biology, University of Copenhagen, Ole Maaløesvej 5, 2200 København N, København, Denmark

## Abstract

Transient antibiotic treatment typically eradicates most sensitive bacteria except a few survivors called persisters. The second messenger (p)ppGpp plays a key role in persister formation in *Escherichia coli* populations but the underlying mechanisms have remained elusive. In this study we induced (p)ppGpp synthesis by modulating tRNA charging and then directly observed the stochastic appearance, antibiotic tolerance, and resuscitation of persister cells using live microscopy. Different physiological parameters of persister cells as well as their regularly growing ancestors and sisters were continuously monitored using fluorescent reporters. Our results confirmed previous findings that high (p)ppGpp levels are critical for persister formation, but the phenomenon remained strikingly stochastic without any correlation between (p)ppGpp levels and antibiotic tolerance on the single-cell level. We could not confirm previous notions that persisters exhibit markedly low concentrations of intracellular ATP or were linked to post-transcriptional effects of (p)ppGpp through the activation of small genetic elements known as toxin-antitoxin (TA) modules. Instead, we suggest that persister cell formation under regular conditions is driven by the transcriptional response to increased (p)ppGpp levels.

## Introduction

Phenotypic heterogeneity is a common feature of clonal bacterial populations due to the noise of intracellular processes^1^, the function of regulatory architectures^2^, and fluctuations in microenvironments. Consequent differences in the behavior of individual cells can increase the fitness of the population because they allow a fraction of cells to be pre-adapted for future changes in the environment, a phenomenon known as bet-hedging^3,4^. Phenotypic heterogeneity can also help bacteria to avoid transient life-threatening situations such as bacteriophage attack^5^ or antibiotic treatment^6^. The latter phenomenon is based on the formation of specialized, antibiotic-tolerant cells called persisters and has been observed in all bacteria investigated, including important pathogens^7,8^. Though the molecular mechanisms underlying persister formation have been intensively studied, no comprehensive understanding of this important phenomenon has been achieved. In most of the suggested models, changes in the levels of nucleotides (ATP, GTP) or nucleotide analogue secondary messengers (cAMP, ppGpp) play key roles^8–11^.

Persister formation of *Escherichia coli* largely depends on the alarmone (p)ppGpp, which controls the stringent response and is also connected to toxin-antitoxin (TA) modules through different pathways^12–14^. TA modules are abundant small genetic elements that can ramp down bacterial growth through dosed activation of toxin proteins upon release of their inhibition by cognate antitoxins^15^. Although ectopic expression of various toxins readily induces antibiotic tolerance^16–18^ and some of the toxins are more potent in the presence of (p)ppGpp^19^, it is not clear if and how many TA modules are involved in persister cell formation under natural conditions. Any kind of bacteriostatic treatment including the expression of toxic proteins impairs antibiotic killing^20,21^. Furthermore, a well-studied mutant allele of the *hipBA* TA module was found in clinical isolates and causes a high persister phenotype, but at the same time compromises the activity and toxicity of the HipA toxin^17,22^.

One difficulty of studying persister formation is that these cells are present only at very low levels in non-stressed populations during unconstrained growth in rich laboratory media^8^. However, different stress conditions including DNA damage or nutrient starvation induce higher rates of persister formation and are therefore often specifically studied in the field^8–10,13,23^. Such stress conditions are common in the natural environments of bacteria, making these conditions a highly relevant field of study^24^.

Persisters are clearly different from cells that simply cease to grow because of severe stress or lack of nutrients^25,26^: Growth inhibition due to stress or starvation is deterministic and homogeneous throughout the population as well as immediately abolished once the cause of growth inhibition has been removed. Conversely, both entry into and exit from the persister state are stochastic events, but the determinants of this stochasticity are largely unknown^27,28^. We therefore explored the formation of *E. coli* persisters upon induction of (p)ppGpp signaling in response to amino acid starvation, a physiological setup that is known to ramp up persister formation and has been well-studied with regard to other aspects of bacterial cell biology^8,25^. Under stress conditions, the stochasticity of persister cell formation can be caused (i) by the heterogeneity of cellular (p)ppGpp levels among different cells in the population or (ii) by the molecular noise in the regulatory circuit that connects the (p)ppGpp level and the gene expression program controlling the phenotypic transition into the persister state. To distinguish these possibilities, we induced (p)ppGpp signaling in *E. coli* by limiting tRNA charging and followed the sequence of birth, antibiotic survival, and resuscitation of persister cells directly by live microscopy. Furthermore, we correlated these processes with TA module activation, ATP levels, and (p)ppGpp levels at the same time in single cells. We could not confirm previous notions that persister cells exhibited markedly high (p)ppGpp or low ATP concentrations. Although persister formation was often preceded by TA module activation, we did not observe a critical role of previously implicated TA modules in persister formation.

## Results

### Curtailing valyl-tRNA charging strongly stimulates persister formation

Many bacteria including the model organism *Escherichia coli* K-12 MG1655 respond to amino acid starvation with the production of (p)ppGpp to induce a well-studied physiological program known as the stringent response^25^. In order to enable controlled induction of stringent response, we introduced the temperature-sensitive allele of *valS* (*valS*^ts^) into the *E. coli* K12 reference strain MG1655 (SEM3147). This mutant exhibits increased intracellular (p)ppGpp levels at elevated temperatures due to the limited activity of L-valyl tRNA synthetase^29^. As expected, the growth of this strain was completely inhibited at 42 °C but cells could resume growth at 30 °C after overnight incubation at 42 °C (Fig. S1A). We observed a strong elevation of ppGpp levels upon changing the growth conditions from completely permissive (30 °C) to semi-permissive (37 °C). About 10 minutes after the temperature shift, the intracellular ppGpp concentration increased approximately 16-fold compared to the level at 30 °C. At 80 minutes, the levels were still approximately 9-fold higher (Fig. S1B).

**Figure 1:**
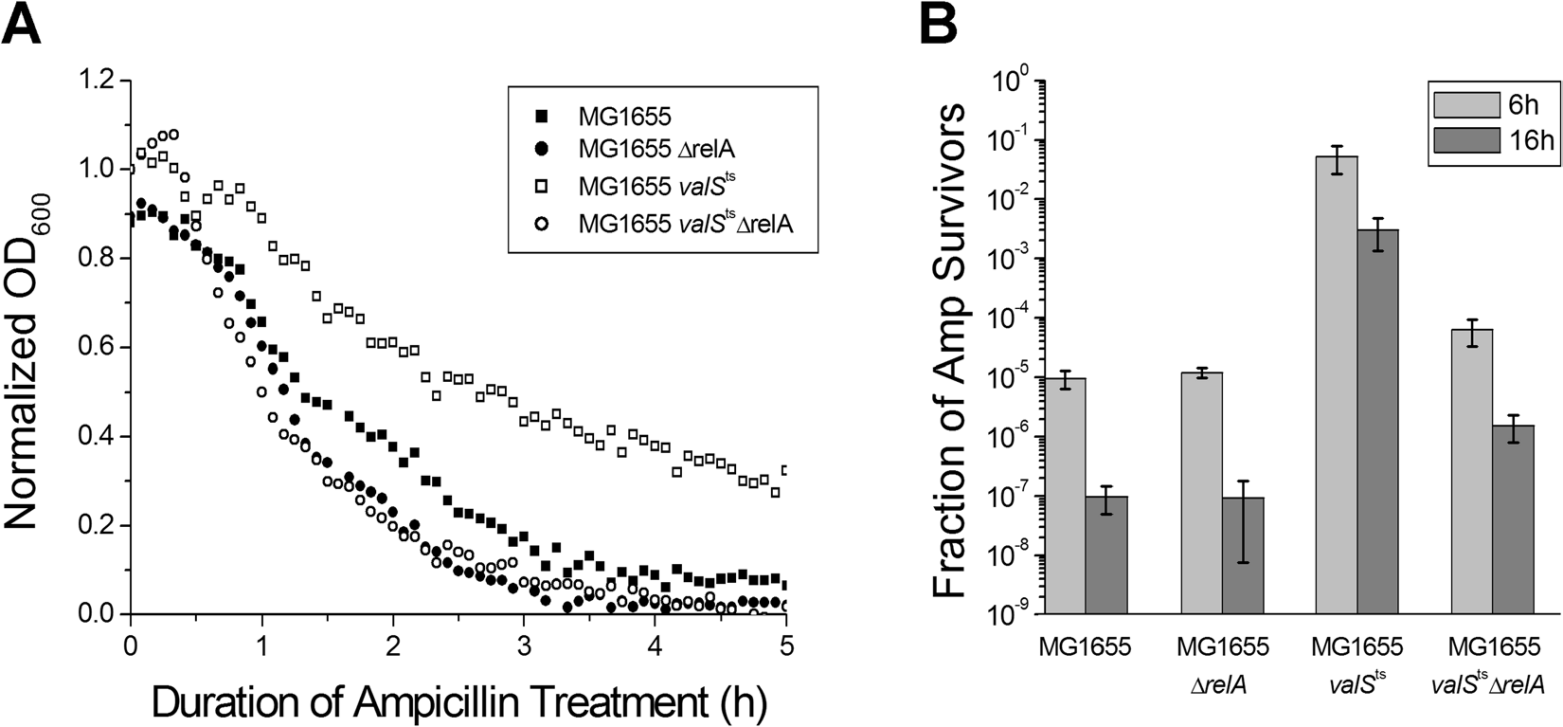
RelA dependent persister cell formation induced by limitation of tRNA charging. Stationary phase MG1655*valS*^ts^ cells were diluted 1:1000 and grown under semi-permissive conditions (at 36.6 °C) until OD_600_ ~ 0.1 in a temperature-controlled microtiter plate reader, in supplemented M9 medium. MG1655, MG1655Δ*relA*, and MG1655Δ*relAvalS*^ts^ cells were used as controls. (**A**) Cells were treated with 150 μg/ml ampicillin, and the optical density of the cultures was recorded over time. The continuous decrease of OD_600_ over the 16 h incubation with ampicillin indicates that no resistant mutants appeared in the samples. (**B**) Colony forming units were determined before and at different time points during treatment; the ratios of survivors after 6 h and 16 h of treatment were plotted.

In order to test whether partial inhibition of ValS activity and the consequent increase of (p)ppGpp affected the frequency of persister cell formation, we grew exponentially growing cultures of MG1655*valS*^ts^ under semi-permissive conditions (36.6 °C) and challenged the bacteria with a lethal dose of ampicillin. The *valS*^ts^ strain formed 3 to 4 orders of magnitude higher levels of antibiotic-tolerant cells than cultures of the control strains, MG1655, MG1655Δ*relA*, and MG1655Δ*relAvalS*^ts^, but still exhibited a majority of antibiotic-sensitive cells (Fig. 1). These results show that impaired tRNA charging increases the rate of stochastic persister cell formation in a way dependent on RelA, the (p)ppGpp synthetase that is known to respond to uncharged tRNA and thus amino acid starvation^25^. This result is similar to previous work showing that the formation of antibiotic-tolerant cells remained a stochastic phenomenon even upon ectopic expression of toxins, because the majority of cells invariably remained antibiotic-sensitive^18,19,30,31^. We therefore conclude that *E. coli* MG1655*valS*^ts^ is a suitable model to study the stochasticity of (p)ppGpp-dependent persister formation, but different from previous approaches in that high levels of persister cells are achieved without ectopic gene expression.

### Construction of a system for single cell analysis of persisters

In order to address the source of stochasticity in persister formation, we developed a system which enables the analysis of persister formation, survival of antibiotic treatment, and resuscitation at single cell level using fluorescence microscopy. To this end, we allowed *E. coli* MG1655*valS*^ts^ cells to develop micro-colonies from single cells under semi-permissive conditions in the microscopy chamber, treated the bacteria with ampicillin, and allowed resuscitation of the antibiotic-tolerant survivors after removal of the antibiotics and stress. This experimental system allowed us to trace the history of cells that could resuscitate after ampicillin treatment by comparatively analyzing the development of (p)ppGpp levels, TA promoter activities, and ATP concentrations in persister and non-persister cells using fluorescent markers. Rising (p)ppGpp levels in individual cells were detected as increased fluorescence from an RpoS-mCherry fusion that is commonly used as a (p)ppGpp reporter in the field^14,32–34^. As expected, incubation of MG1655*valS*^ts^ cells carrying the chromosomal RpoS-mCherry fusion under non-permissive conditions (41 °C) showed a strong induction of fluorescence (~10-fold; Fig. S1C).

In order to study the induction of TA modules, we chose the RelBE system as a model because its regulation is well understood^35–37^ and it was previously shown to be activated by amino acid starvation like a number of other TA modules of *E. coli* K12^11^. Activity of the RelBE module is regulated through the stability of the RelB antitoxin. Destabilization of RelB by Lon-mediated proteolysis^35^ results in derepression of the *relB* promoter and accumulation of the RelE toxin^37,38^. As a reporter for RelBE activation, we therefore used a short lived YFP (or mCherry) variant, here called YFP^unstable^ (mCherry^unstable^)^39^, transcribed from the *relB* promoter. Consistent with previous work, the *relB* promoter was robustly repressed by transcriptional autoregulation in *E. coli* K-12 wildtype cells^35^ but showed strong expression in a Δ*relBE* mutant (Fig. S1D).

ATP levels in individual cells were monitored using the ATP sensor QUEEN-7µ^40^ which was suitable to determine ATP concentrations in the physiologically relevant range of ~0.05–10 mM^40^ in our setup (Fig. S1E). Unlike the (p)ppGpp and TA reporters, which are limited by the time scale of the correlation of the signal and protein level changes, the ATP reporter shows the actual ATP concentration in cells as it is based on an ATP-induced conformational change in the reporter protein.

### Full time course of stochastic formation and resuscitation of persisters with high toxin-antitoxin transcription in the stressed population

In order to analyze the development of (p)ppGpp levels and TA promoter activities in single cells exposed to limitation of tRNA charging, MG1655 *valS*^ts^ cells carrying the chromosomal RpoS-mCherry fusion and the plasmid-borne *relB* promoter-YFP^unstable^ fusion were studied by fluorescence microscopy. Cells were grown without limitation of tRNA charging before microscopy and then shifted to semi-permissive conditions (36.6 °C) in the microscopy chamber. Importantly, the majority of cells were able to grow at these semi-permissive conditions and micro-colonies containing up to ~150 cells developed from single cells over 17 hours. These micro-colonies were treated with ampicillin for 10 h which caused lysis and death of most cells. Subsequently, the drug was removed by addition of purified β-lactamase and resuscitation of antibiotic-tolerant survivors was enabled by restoring tRNA charging via a temperature shift to permissive conditions, and the cells were incubated for additional 13 h.

During the growth of micro-colonies at semi-permissive temperature for the *valS*^ts^ allele, a few cells became non-growing and remained intact upon exposure to ampicillin. When the antibiotics were removed, some of the cells started to grow and successfully divided during the 13 h of subsequent incubation. We consider these cells as persisters in the present analysis. Note that these persister cells are not resistant cells, since they did not grow in the presence of antibiotics. In addition, there were also cells that stayed intact over the full time course but did not grow after the removal of antibiotics within the incubation time. Due to the technical limitations of the experimental setup, we could not draw a conclusion about the fate of these cells. Here, we call them dormant cells and distinguish them from the persister cells. Furthermore, we observed another class of cells as well that survived the ampicillin treatment in a cell wall deficient L-form-like state^41^ (Fig. S2A).

**Figure 2:**
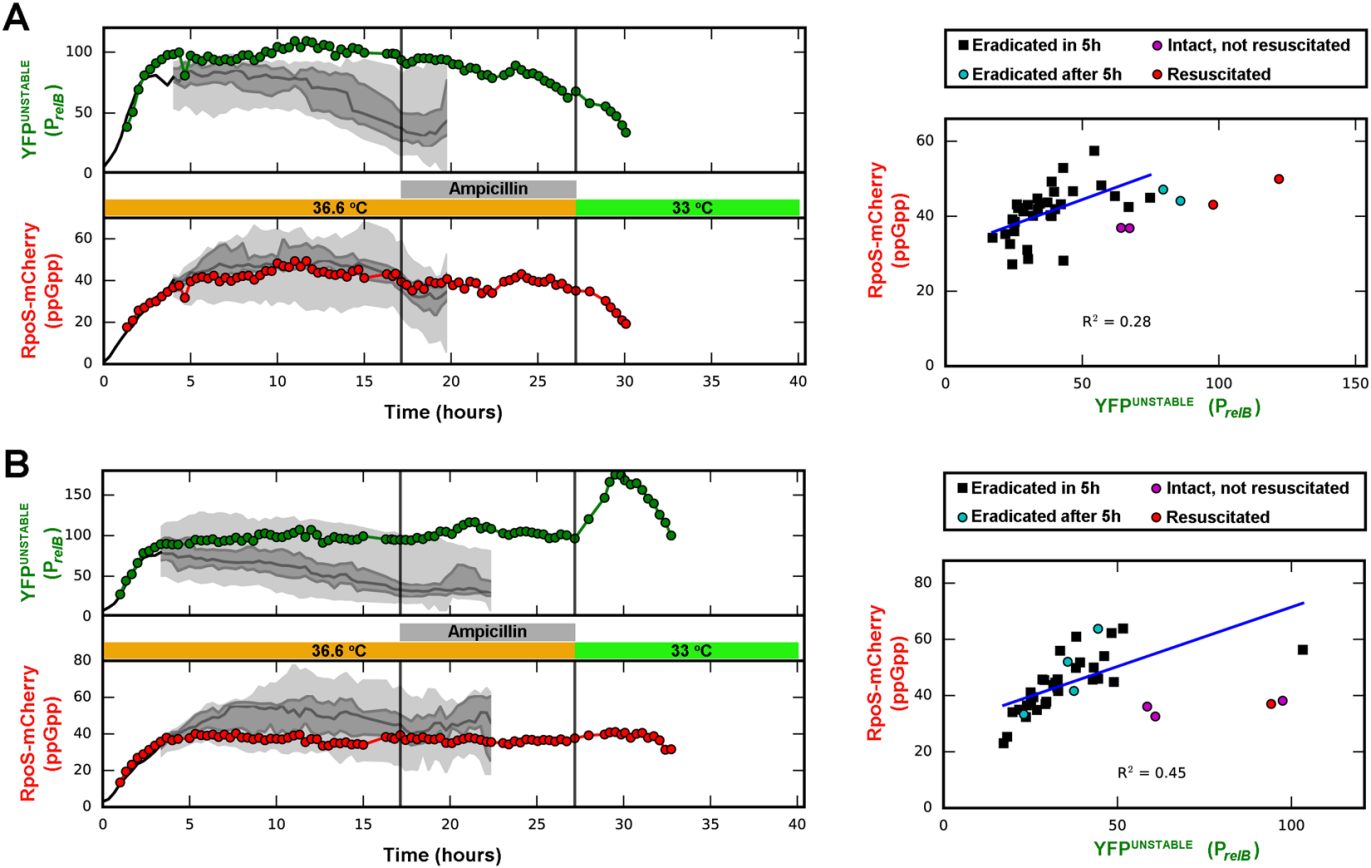
Single cell analysis of persister cell formation, ampicillin survival, and resuscitation in two micro-colonies (**A**,**B**). Left panels: MG1655*valS*^*ts*^*rpoS::mcherry* cells carrying the plasmid borne *relB* promoter-*yfp*^unstable^ fusion (pSEM4102) were grown in a temperature controlled microscope at semi-permissive temperature (36.6 °C). Micro-colonies that developed from single cells were treated with ampicillin. After ampicillin treatment, the antibiotic was removed and the temperature was lowered to 33 °C, allowing resuscitation of the antibiotic survivors. Fluorescence of *relB* promoter (YFP^unstable^) and the (p)ppGpp (RpoS-mCherry) reporters was recorded for all cells. Traces for two persister cells are shown in color. The traces end at first division after resuscitation, and the last data point represents the mean fluorescence of the two progeny cells after division. The median values of fluorescence are plotted along with the 25th, 50th, 75th and 100th percentiles of the non-persister cells of the colony. The percentiles are shown when there are 8 or more cells in the colony. Right panels: Distribution of fluorescence levels in the cells of the corresponding micro-colonies right before addition of ampicillin. The linear fit represents the correlation of the two markers in the non-persister population. More trajectories and corresponding pre-ampicillin distributions are shown in Fig. S2B. M9 medium supplemented by 3.2% glycerol and 17 amino acids (0.1 mg/mL) were used.

Figure 2 shows the development of mCherry and YFP^unstable^ fluorescence for individual bacteria as well as for the whole population of two microcolonies. Both sensors were substantially induced upon amino acid starvation, but displayed large cell-to-cell heterogeneity.

Interestingly, the population of cells surviving antibiotics had significantly higher YFP^unstable^ fluorescence than the susceptible population in the last measurement before addition of antibiotics (3-fold increase in mean, p << 0.001, Mann-Whitney test), indicating that TA module activation is directly or indirectly linked to persister formation (Figs 2, S2B). Importantly, the life time of the YFP^unstable^ was about 100 min in the stressed condition in the non-persister cells, while the fluorescence stayed high for 10 hours or more in the persisters. That is, although the persister cells were in a growth-arrested state, they maintained high levels of YFP^unstable^, most likely due to active transcription of the *relB* promoter and active global translation.

Resuscitation of persister cells coincided with strong repression of the *relB* promoter, although we found two distinct trajectories of reporter levels in the persisters observed. In Fig. 2A, the level of the *relB* promoter reporter started to decrease as soon as the stress was removed. However, in Fig. 2B, the reporter activity first started to increase upon removal of stress and then decreased before the resuscitated cell divided. The resuscitation trajectories for other persisters exhibit similar features (Fig. S2B).

### RelB promoter activation through (p)ppGpp and Lon-dependent antitoxin degradation

Our results that amino acid starvation causes an induction of RpoS-mCherry fluorescence (representing (p)ppGpp levels) throughout the population as well as a concomitant increase in the frequency of antibiotic-tolerant cells were not unexpected (Fig. 2). However, despite the correlation of these phenomena on the population level, the intensity of RpoS-mCherry induction of single cells was clearly not predictive of persister formation: Cells with higher or lower levels of reporter fluorescence were eradicated by ampicillin with similar chances (Fig. 2, right panels). The stochasticity of persister formation was therefore not rooted in heterogeneous (p)ppGpp levels but must instead be inherent to the signaling downstream of high (p)ppGpp in individual cells.

**Figure 3:**
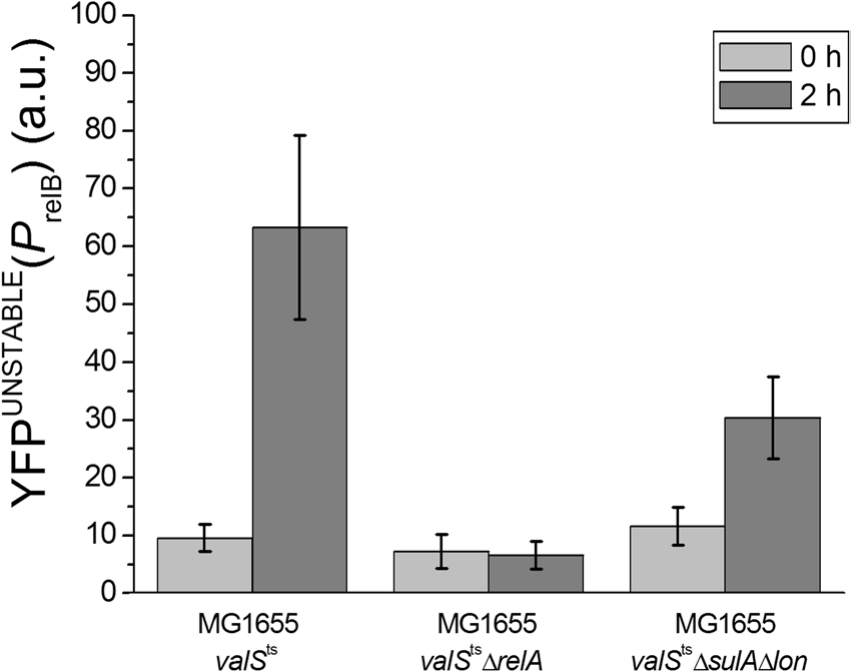
Changes in the levels of the *relB* promoter reporter (YFP^unstable^) upon limitation of tRNA charging in MG1655*valS*^ts^, MG1655*valS*^ts^Δ*sulA*Δ*lon*, and MG1655*valS*^ts^Δ*relA* cells carrying the plasmid borne *relB* promoter-*yfp*^unstable^ fusion (pSEM4102). Cells were grown in a temperature-controlled microscope on supplemented M9 agarose pads as described in Fig. 2. Images were taken at 0 time and after 2 hours growth at 36.6 °C. Similar results were obtained upon treatment with the seryl-tRNA synthetase inhibitor serine hydroxamate (SHX) at permissive temperature, indicating that they are not linked to the MG1655*valS*^ts^ strain or temperature stress but truly reflect the biology of limited tRNA charging (Fig. S3A).

This result was surprising to us, because previous work had shown that the RelBE module is activated through degradation of the RelB antitoxin in response to increasing (p)ppGpp levels in a way dependent on the Lon protease^35^. We had therefore intuitively assumed a dose-dependent effect of (p)ppGpp on RelB degradation and RelBE activation. In order to confirm that RelBE activation in response to limitation of tRNA charging indeed occurred via this pathway and required (p)ppGpp as well as Lon, we compared activation of the *relB* promoter in MG1655 *valS*^ts^, MG1655 *valS*^ts^Δ*lon*, and MG1655 *valS*^ts^Δ*relA* cells. Bacteria were grown like in the experiment presented in Fig. 2, and YFP^unstable^ fluorescence was measured at 0 time and after 2 hours under semi-permissive conditions (at 36.6 °C). Figure 3 shows the changes of fluorescence levels.

As expected, a strong induction of RelBE upon limitation of ValS activity was observed with *E. coli valS*^ts^ but not the *valS*^ts^Δ*relA* mutant that is unable to synthesize (p)ppGpp in response to amino acid starvation (Fig. 3). Similar results to these experiments with a transcriptional P*relB::yfp*^unstable^ fusion were obtained with an analogous translational RelB-YFP^unstable^ fusion (Fig. S3). Interestingly, *E. coli valS*^ts^Δ*sulA*Δ*lon* cells showed an intermediate activation of RelBE expression (Fig. 3). This observation confirms that the signaling from (p)ppGpp synthesis to RelB degradation indeed occurs primarily through the Lon protease^32,35,42^ but also suggests that there can be an additional alternative pathway.

### Intracellular ATP concentrations are not correlated with antibiotic survival upon induction of (p)ppGpp synthesis

**Figure 4:**
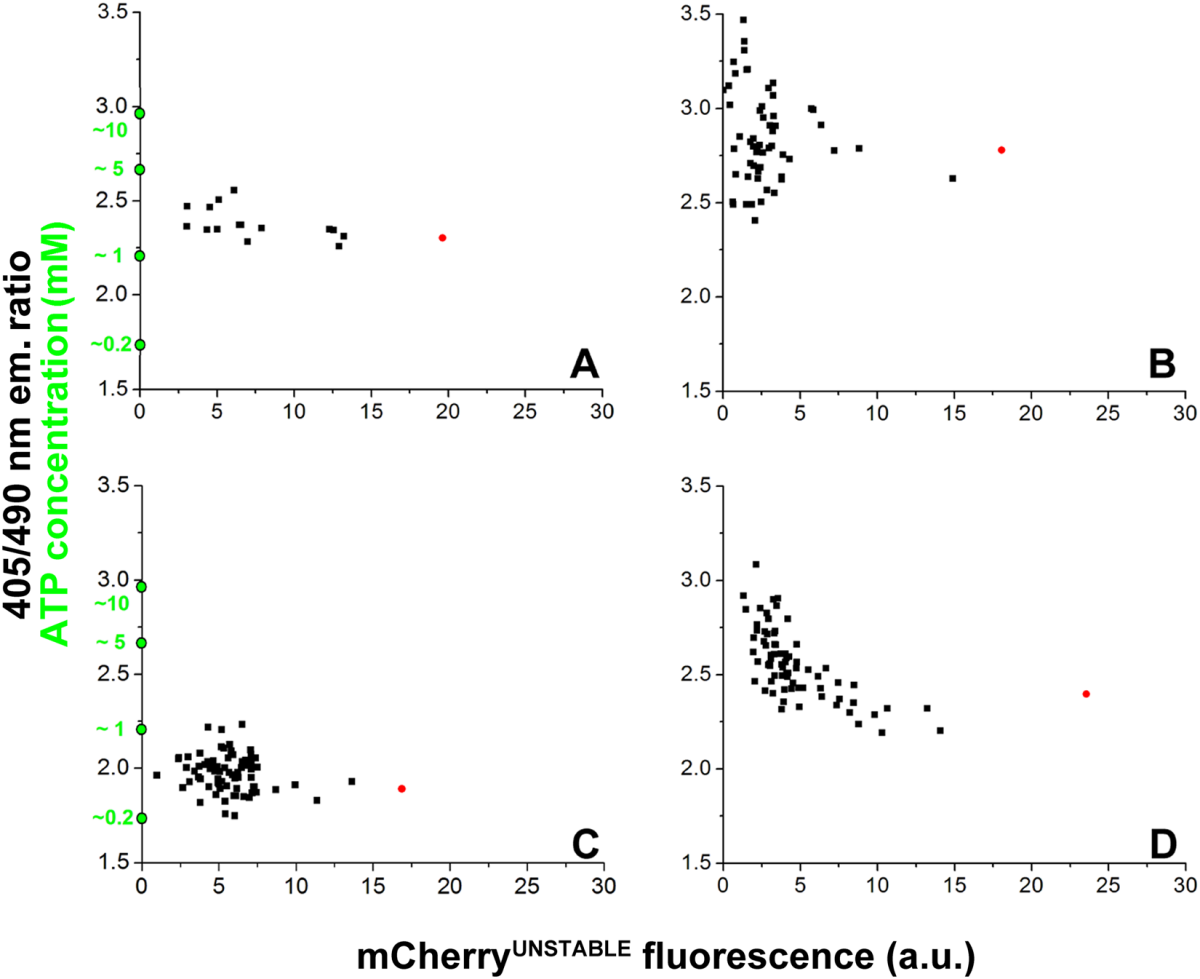
Intracellular ATP concentration is not correlated with (p)ppGpp induced persistence. *E. coli* MG1655*valS*^ts^ cells carrying the pSEM4157 plasmid were grown in a temperature-controlled microscope as described for the experiment presented in Fig. 2. The dynamics of the *P*_relB_ promoter were followed by measuring the fluorescence of the mCherry^unstable^ protein, transcribed from the *P*_relB_ promoter in plasmid pSEM4157. The intracellular ATP concentration was determined from the QUEEN-7µ fluorescence by calculating the ratio of the emitted light intensities at 520 nm using 405 and 490 nm excitation wavelengths^40^. To follow the development of micro-colonies from single cells and appearance of persisters, QUEEN-7µ and *P*_relB_-mCherry fluorescence levels were recorded every 20 minutes. Colonies were allowed to develop for 15 h and were then treated with ampicillin for 10 h. After removal of ampicillin, survivor cells were allowed to grow under permissive conditions (at 33 °C). Cells that were able to divide within 13 h were considered persisters. ATP levels and the level of the *P*_relB_-*mCherry*^unstable^ reporter were analyzed in colonies before ampicillin treatment (A–D). Red dots correspond to persister cells. ATP concentrations were calculated as described in *Materials and Methods*. The development of ATP and mCherry^unstable^ levels during resuscitation of persisters is shown in Fig. S4.

The intracellular concentration of ATP is a major determinant of cellular physiology and known to be linked to bacterial persistence in several ways. On one hand, persister formation can be enhanced by ramping down cellular processes due to low ATP concentrations^11^. However, maintaining sufficiently high ATP is important for the antibiotic tolerance of other types of persister cells because it fuels active efflux processes^43–45^. To analyze the links between persister formation, ATP concentration, and TA module activation, we constructed a plasmid that carries the ATP sensor QUEEN-7µ^40^ and the mCherry^unstable^ protein, transcribed from the *relB* promoter (pSEM4157). Growth, survival of antibiotic treatment, and resuscitation of *E. coli valS*^ts^ cells carrying the pSEM4157 plasmid were studied by fluorescence microscopy at semi-permissive conditions similar to the experiment shown in Fig. 2. We did not detect any significant correlation between *relB* promoter activity and intracellular ATP concentration, indicating that these two parameters are not directly linked (Fig. 4). Retrospective analyses of colonies (formed from single cells) before ampicillin treatment confirmed our previous observation that persister cells displayed higher levels of TA module activation (mCherry^unstable^ fluorescence) than their non-persister peers (Fig. 4; compare to Fig. 2). However, we did not observe that their ATP concentrations were markedly different (Fig. 4).

In the resuscitation, the level of the *relB* promoter reporter shows the two distinct trajectories as discussed before (Fig. S4; compare to Fig. 2). The ATP levels also showed different trajectories during resuscitation but did not correlate with the reporter of the *relB* promoter activity. Also, there was no correlation between the ATP level at the time of ampicillin removal and the time required for the first doubling.

### Resuscitation of persisters correlates with cessation of toxin-antitoxin transcription

**Figure 5:**
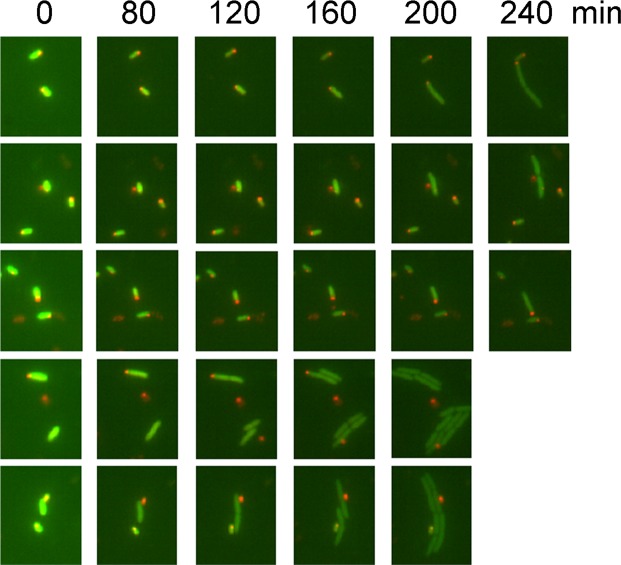
Resuscitation of persisters is correlated with cessation of *relBE* transcription. MG1655*valS*^*ts*^*rpoS::mcherry* cells carrying the pSEM4102 plasmid were grown in LB medium at 37 °C for about 6 hours (to mid-log phase) and treated with 100 µg/ml ampicillin for 3 hours, also at 37 °C. Survivor cells were concentrated by centrifugation and placed on a supplemented M9 agarose pad that contained β-lactamase to degrade any remaining ampicillin carried over from the growth medium. Changes in the cellular levels of YFP^unstable^ and RpoS-mCherry were followed by fluorescence microscopy at 33 °C. Images were taken at the indicated time points. Combined mCherry (red) and YFP (green) channels are shown. Quantification of mCherry and YFP^unstable^ fluorescence for 28 individual cells is shown in Fig. S5.

To obtain more insight into the resuscitation process, we grew MG1655*valS*^*ts*^*rpoS::mcherry* cells carrying the pSEM4102 plasmid in a batch culture under semi-permissive conditions (37 °C) to mid-log phase and treated the culture with ampicillin. Changes in the cellular levels of YFP^unstable^ and RpoS-mCherry during resuscitation of the survivor cells were followed by fluorescent microscopy under permissive conditions (at 33 °C; Fig. 5).

As expected from our previous experiments (Figs 2 and S4), cells that survived the ampicillin treatment displayed high YFP^unstable^ levels (Fig. 5). However, YFP^unstable^ levels rapidly decreased as ValS activity was restored due to the shift to permissive temperature. Importantly, YFP^unstable^ levels always decreased before the first cell division, indicating abrogation of RelB antitoxin degradation (resulting in RelE toxin inhibition) and restoration of *relBE* auto-repression during resuscitation (Fig. S5). Similar to the other resuscitation experiments (Figs 2 and S4), both RpoS-mCherry and YFP^unstable^ fluorescence showed variable trajectories during the resuscitation process (Fig. S5). In all the three resuscitation experiments, cells resumed growth stochastically, and not all the cells that had remained intact after the ampicillin treatment went through a cell division during the experiment.

### Limited tRNA charging induces persister formation in the absence of mRNase toxins

**Figure 6:**
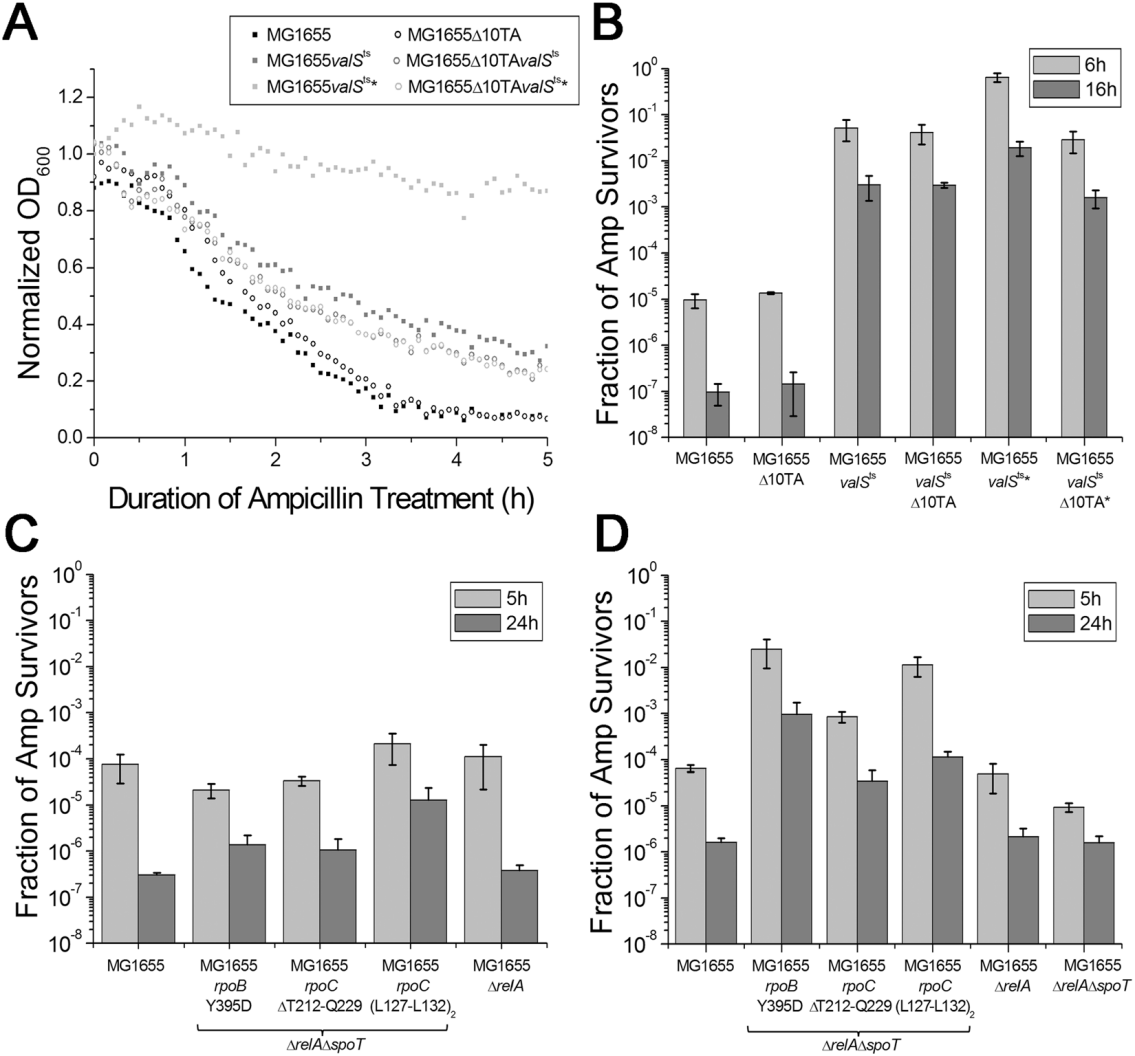
(**A**,**B**) Persister cell formation upon limitation of tRNA charging in the strain lacking the 10 mRNase toxins (Δ10TA). Stationary phase MG1655*valS*^ts^ cells were diluted 1:500 and grown at 36.6 °C until OD_600_ ~ 0.1 in a temperature-controlled microtiter plate reader. MG1655, MG1655Δ10TA, and MG1655*valS*^ts^Δ10TA cells were used as controls. Cells were treated with 150 μg/ml ampicillin, and the optical density of the cultures was recorded over time (**A**). Colony forming units were counted before the treatment and 6 h and 16 h after treatment (**B**). Cells were treated with β-lactamase before plating on LB plates. Cultures marked with a star were shifted to room temperature for 5 minutes after 4 h of incubation and incubated for an additional ~2 h (until they reached OD_600_ ~ 0.1) before ampicillin treatment. (**C**,**D**) Antibiotic survival of MG1655 Δ*relA* Δ*spoT* stringent mutants in supplemented M9 medium (**C**) or LB medium (**D**). Stationary phase cultures of the indicated strains were diluted 1:100 into fresh medium and directly challenged with 100 μg/ml ampicillin. Colony forming units were determined over time for 24 h, revealing biphasic killing. Bar diagrams display average and standard deviation of bacterial survival after 24 h of treatment. The growth of all strains in both media is shown in Fig. S6.

Our results showed that persister formation was correlated with activation of the RelBE TA module (Fig. 2), indicating that the two events are linked. RelBE is one of ten TA modules in the genome of *E. coli* K-12 that encode toxins with mRNA endonuclease (mRNase) activity and inhibit bacterial growth by globally inhibiting translation through mRNA depletion^15^. Others had previously shown that the whole set of ten mRNase TA modules is activated upon amino acid starvation, suggesting that they might be at least partially redundant^11^. We therefore used a mutant lacking all ten mRNase toxin genes, *E. coli* Δ10TA^46^, to test whether the increased rate of persister cell formation upon limitation of tRNA charging depended on mRNase TA modules. Wild type and Δ10TA cells were grown to mid-log phase and treated with ampicillin as described before to score the formation of antibiotic-tolerant persisters (Fig. 6). As expected from previous results with different Δ10TA variants^11,46^, we did not see any difference between wildtype *E. coli* and the Δ10TA mutant (Fig. 6, MG1655 and MG1655Δ10TA). We therefore conclude that the basal rate of persister formation in unstressed *E. coli* does not depend on the set of ten mRNase TA modules. Subsequently, we performed the same experiment with the *valS*^ts^ variants of the wild type and Δ10TA strains under semi-permissive conditions to investigate whether the results would be different under conditions of high (p)ppGpp levels that should result in activation of the TA modules. Again, we did not detect any significant difference between the wildtype and the Δ10TA of *E. coli valS*^ts^ (Fig. 6, MG1655*valS*^ts^ and MG1655*valS*^ts^Δ10TA).

In retrospect, this result was not unexpected, given that high (p)ppGpp is known to not only promote the degradation of TA module antitoxins in *E. coli* K-12^35^ but also to globally ramp down gene expression^25^. A simple, single pulse of antitoxin degradation might therefore not be sufficient for a phenotypic switch into the persister state if levels of pre-existing toxin proteins are too low. We therefore changed the experimental procedure to include a short (5 minutes) incubation at permissive temperature after 4 hours of the 6 hours growth under semi-permissive conditions (36.6 °C). In this setup, the number of persister cells further increased around tenfold in *E. coli valS*^ts^ cells but not in *E. coli valS*^ts^Δ10TA cells (Fig. 6, MG1655*valS*^ts^★ and MG1655*valS*^ts^Δ10TA★).

### Transcriptional effects of the stringent response are sufficient for wildtype levels of persister formation in the absence of (p)ppGpp

Our results confirm previous notion that basal levels of (p)ppGpp (maintained by SpoT in the *relA* mutant) are sufficient to generate wildtype levels of persister cells under unstressed conditions (Figs 1 and 6)^30^. Conversely, a complete lack of (p)ppGpp in a mutant lacking both *relA* and *spoT* causes an increased sensitivity to drug treatment and decreased levels of persister cells particularly in minimal media^19,46^. Because a single stress event resulted in similar increase in the presence and absence of the 10 mRNase TA systems, we wondered whether persister cell formation is regulated solely by (p)ppGpp mediated reprogramming of promoter recognition by RNA polymerase, or if it requires post-transcriptional effects of the alarmone such as allosteric or competitive regulation of other protein functions^25^. We therefore selected a number of “stringent mutants” of Δ*relA*Δ*spoT* cells with mutations in the RNA polymerase genes *rpoB* and *rpoC* that, unlike the parental strain, readily grow in minimal media in the absence of any (p)ppGpp due to activation of the stringent transcriptional program^47–49^. For further experimentation, we chose two different mutants (#5 with RpoC ΔT212-Q229 and #6 with duplication of L127-L132 in RpoC) that grew similar to wildtype in supplemented M9 medium and another one (#1 with RpoB Y395D) that grew slower (Fig. S6).

We found that all three stringent mutants displayed persister levels that were at least as high as the wildtype in M9 medium, or even much higher in LB (Fig. 6C,D). Notably, the results obtained with the three stringent mutants were similar and consistent despite their significant differences in growth rate, indicating that the phenotypes were truly due to activation of the stringent transcriptional program.

## Discussion

### Limitation of tRNA charging induces stochastic formation of persister cells through activation of the stringent transcriptional program

In this work we used single cell and population level approaches to analyze physiological parameters of antibiotic-tolerant persister cells forming in response to induction of the stringent response by limitation of tRNA charging. As expected, the partial limitation of valyl-tRNA charging caused *E. coli* to develop high levels of (p)ppGpp (Figs 2, S1B) and resulted in a “responsive diversification” of the population^50^, leading to an elevated rate of persister cell formation (Fig. 1). A remarkable heterogeneity of (p)ppGpp levels was observed in single cells that was not correlated with the chance of differentiation into a persister (Fig. 2). These results demonstrate that high (p)ppGpp levels beyond a certain threshold promote the formation of antibiotic-tolerant cells but that the stochasticity of persister formation is not directly controlled by (p)ppGpp, e.g., in a dose-dependent manner. Instead, we suggest that the reprogramming of transcription contributes to the persister formation, which needs to be triggered by some molecular noise. The need of the reprogramming of transcription is supported by the observation that the rate of persister cell formation can be regulated by mutations in the RNAP subunits in the physical absence of (p)ppGpp (Fig. 6C,D). The stochasticity of the persister formation supports the importance of the molecular noise, which by chance helps a cell to go over the “barrier” between the ordinary transcription state and the reprogrammed state.

In our experimental system, formation of persister cells was solitary, that is, there was no obvious correlation between the fate of the persister cell and its sister (Fig. S2C). As persister cells developed from cells having either old or young poles without obvious preference, the asymmetry in cell fates was not due to the aging cell poles^51–53^, which correlated with antibiotic tolerance in other kinds of stress conditions^54,55^.

### Persister cells formed in response to limited tRNA charging do not display particularly low ATP levels

ATP depletion was recently found to be the main mechanism of persister formation in *Staphylococcus aureus*^56^. Follow-up work later showed that artificial reduction of ATP levels in *E. coli* cells increased antibiotic tolerance^11^. In the current study, we directly monitored the intracellular concentration of ATP in a more natural model of stress-induced persister formation. Our results show that the limitation of tRNA charging and consequent activation of (p)ppGpp signaling resulted in a decreased average level of intracellular ATP in the population (Fig. 4), though with a large heterogeneity as had also been reported for non-stressed *E. coli* before^40^. In our setup, we did not detect any correlation between intracellular ATP levels and the chance of individual cells to survive ampicillin treatment (Fig. 4). These findings might differ from the model proposed by Shan *et al*. because these authors did not study ATP levels of native persister cells but instead merely reported on the effect of pharmacologically interfering with ATP homeostasis of *E. coli*^11^.

Interestingly, the persister cells observed by fluorescence microscopy in this study maintained high levels of YFP^unstable^, supporting the emerging view that persister cells can be metabolically active and synthesize specific proteins such as drug efflux pumps that help them cope with high antibiotic concentrations^31,44,57^.

### Persister cell formation in response to limited tRNA charging correlates with TA module activation

The cells responded to elevated (p)ppGpp levels in our assay setup with increased levels of the reporter for TA module activation, and persister cells had even higher levels of the reporter compared to their regularly growing clonal peers (Figs 2 and 4). We confirmed that activation of the *relB* promoter required the presence of starvation-induced (p)ppGpp synthetase RelA (Fig. 3), which was also required for fast degradation of the RelB antitoxin (Fig. S3). These observations are in line with the previously proposed regulation of *relB* transcription through proteolytic degradation of RelB^37,58^. In non-stressed cells the *relB* promoter is strongly repressed by RelB_2_RelE antitoxin-toxin complexes. Fast degradation of RelB in the presence of stress results in lower RelB:RelE ratios, where RelB_2_RelE_2_ complexes dominate. These complexes are not able to bind the *relBE* operator and repress *relBE* transcription due to a steric clash between the additional RelEs present in the tetramers^38^. The signaling from RelA activation to RelB degradation occurs primarily through the Lon protease^35^ but an alternative pathway might exist which is active in the absence of Lon (Fig. 3). This alternative pathway can either be an additional protease activated by the amino acid starvation to degrade RelB, or an unknown mechanism of transcription activation of relB, epistatic over RelB repression. The difference of the reporter level between the non-growing persisters and their growing peers are likely to be a combined effect of transcriptional derepression and reporter accumulation due to the lack of dilution by cellular growth.

### TA-dependent and TA-independent formation of persister cells

It was previously proposed that persister cells formed during unconstrained growth of *E. coli* K-12 in response to stochastic peaks of (p)ppGpp through a linear pathway composed of polyphosphate accumulation and the degradation of antitoxin proteins by the Lon protease, resulting in mRNase TA module activation^59^. However, we recently discovered that the results underlying this model of persister formation were affected by a number of artifacts including phenotypic changes due to inadvertent lysogenization of mutant strains with the ϕ80 prophage^46^. Most importantly, we demonstrated that a reconstructed mutant strain devoid of all ten mRNase toxins did not display any defect in persister cell formation during unconstrained growth^46^.

Nevertheless, we find that induction of (p)ppGpp signaling in response to impaired tRNA charging results in (i) increased rate of persister cell formation (Fig. 1) as well as (ii) activation of the RelBE TA module (Fig. 2). However, the formation of these stress-induced persister cells was largely independent of RelBE and all other mRNase TA modules (Fig. 6). Note that this is consistent with the previous work that found no difference in persistence between WT and RelBE single mutant cells, even though in WT higher transcription of TA modules including RelBE was observed^20^. Furthermore, the non-growing state of the persister cells formed in our assay setup was mostly maintained by the stress condition and did not require these TA modules (Fig. 6). The likely explanation of these observations is that the *relBE* promoter activity is “downstream” of the persister formation pathway; an event that plays a role in persister formation also increases the *relBE* reporter expression. Further studies are needed to find out what is the direct cause of the *relBE* reporter expression change in persister cells.

When the normal level of tRNA charging was restored, about half of the persister cells responded immediately by (i) decreased (p)ppGpp levels, (ii) shut-down of the *relB* promoter, and (iii) resumed cell division (Fig. S2B). However, a second group of cells reacted much slower, maintained a strong response of the (p)ppGpp reporter for much longer, and displayed a characteristic peak of *relB* promoter expression (Fig. 2A). We speculate that in these cells the levels of toxin protein have crossed the threshold required to inhibit bacterial growth, so that a restoration of regular toxin-antitoxin balance is required before resuscitation. The strong expression of toxins and other toxic proteins is known to be sufficient for inducing antibiotic tolerance^16–18,30^ but our results show that TA module activation in our assay setup is redundant with other stress-induced physiological changes (Fig. 6).

It is possible that mRNase TA modules fail to make a major contribution to stress-induced persister formation because the global inhibition of gene expression by insufficient tRNA charging prevents sufficient *de novo* expression of toxin molecules from the de-repressed TA loci in parallel to antitoxin degradation. Interestigly, a tenfold increase of persister levels was observed with wildtype *E. coli valS*^ts^ but not the Δ10TA variant when we split the time of growth under semi-permissive conditions and introduced a transient temperature downshift (Fig. 6). A possible interpretation of this result is that the transient abrogation of translation inhibition enables the TA module mRNAs expressed during the period of starvation to be translated, resulting in a higher level of toxin-antitoxin protein complexes. This pre-loading of the cell would result in the liberation of much higher amounts of toxin molecules upon next exposure to amino acid starvation which could sustain the phenotypic transition into persistence. This hypothesis is highly speculative, and future experimental studies are needed to uncover the molecular basis of this “memory effect” of persister formation in response to repeated amino acid starvation. Notably, the phenomenon would be superficially similar to the control of persister formation by an unrelated TA module, TisB/*istR-1*, of *E. coli* K-12. Previous work established that the TisB toxin is induced by massive (yet still sublethal) DNA damage and can drive the formation of persister cells that survive subsequent antibiotic treatment^23^. It seems therefore possible that the characteristic regulation of TA modules would restrict their full induction by stochastic stimuli or isolated instances of regular stress, possibly in order to protect bacteria from their futile activation^15^ that would interfere with timely recovery once stress is alleviated. However, reoccurrence of stress during the recovery process might unleash the full activation of TA modules, resulting in formation of hardy persister cells that ensure survival of the bacterial population.

## Materials and Methods

### Strain construction

Strains, plasmids and oligonucleotides used in this study are listed in Table S1. The *valS*^ts^ allele in strain JF858^60^ was identified as a point mutation conferring a single amino acid substitution in ValS (P537L). The mutation was transferred to MG1655 as described below. The *valS*^*ts*^ gene of strain JF858 was PCR amplified using primers ValS1 and ValS_Nco. The amplified DNA fragment was digested with NcoI and BglII and inserted between the *Nco*I and *Bgl*II sites of plasmid pEM7/zeo (Invitrogen). Using the resulting plasmid as a template, part of the *valS* gene (containing the *ts* mutation) and the zeocin resistance of the plasmid was PCR amplified using primers ValS2 and ValSREC and the PCR product was used to introduce the *valS*^ts^ mutation into the MG1655 strain by recombineering^61^. Recombinants were selected on LB plates containing 67 µg/ml zeocin and tested for temperature sensitivity. The mutation responsible for the temperature sensitive phenotype in the *valS* gene was verified by DNA sequencing (Eurofins Genomics) in the selected strain (SEM3147). The *valS*^ts^ mutation was introduced into strain EM477 (MG1655 carrying an *rpoS::mCherry* translational fusion)^32^ and into strains AHK250 (SEM4202) and AHK205 the same way (SEM4178).

Strains SEM3156 and SEM3157 were created from strains MG1655 and SEM3147, respectively, by replacing the *relA* gene with a *cm*^*R*^ cassette. The *cm*^*R*^ cassette of plasmid pRFB122^62^ was PCR amplified using primers RelAupCm and RelAdnCm and inserted into the chromosome by recombineering^61^.

Strain AHK205 (MG1655 *sulA*::FRT Δ*lon rpoS*-mCherry) was generated from MG1655 by P1*vir* transduction of *sulA*::*kan*^*R*^ (from the KEIO collection), flipping out the *kan*^*R*^ marker using pCP20^61^, and scarless deletion of *lon* according to a procedure described previously^63^. The *rpoS*::*mCherry*-*frt*-*kan*^*R*^-*frt* cassette^32^ was PCR amplified and then recombineered into the *sulA*::*frt* Δ*lon* strain. Finally, the *kan*^*R*^ cassette was removed using pCP20 and the resulting locus was sequenced, revealing a single ORF for the RpoS-mCherry fusion protein and a single clean FRT scar downstream of the locus.

### Growth Medium

Luria-Bertani (LB) broth was prepared by dissolving 10 g of tryptone, 5 g of yeast extract, and 10 g of sodium chloride per liter of Milli-Q H_2_O and sterilized by the use of an autoclave.

The supplemented M9 medium used in the persister assays was prepared as described by Harms *et al*.^46^.

The M9-based growth medium for microscopy contained 3.2% glycerol (instead of glucose) and 17 amino acids (instead of casamino acids) with a concentration of 0.1 mg/ml (excluding Phe, Tyr, and Trp to limit background fluorescence). The high concentration of glycerol was chosen to secure a high level throughout the whole experiment. In addition, the medium for microscopy contained 1% agarose. The cells were grown on top of this gel.

### Selection of Stringent Mutants

Stringent mutants were selected in the Δ*relA*Δ*spoT* background by washing small samples of LB medium overnight cultures in sterile PBS and streaking them out on M9 SMG agar plates (supplemented M9 liquid medium with 1.4% of agar, no amino acids, and L-serine, L-methionine, and glycine at 100 g/ml as only source of amino acids). Unlike the parental Δ*relA*Δ*spoT* strain, stringent mutants can form colonies under these conditions of amino acid starvation due to mutations in RNA polymerase genes^21^. Randomly chosen colonies from separate overnight cultures were re-streaked on M9 SMG agar plates and the *rpoBC* locus was sequenced to identify mutations underlying the stringent phenotype.

### Plasmid construction

To construct a plasmid expressing RelB fused to the C terminus of Venus YFP, we PCR amplified the DNA fragments encoding RelB and Venus YFP separately, using primers RelYdn and RelYJ2 for RelB, and RelYup and RelYJ1 for Venus YFP. We used MG1655 chromosomal DNA and plasmid pSEM3131B^39^ as templates, respectively. The fragment encoding RelB was cut by *Bam*HI and *Kpn*I, while the fragment encoding Venus YFP was cut by *Pst*I and *Kpn*I. The two fragments were inserted between the *Pst*I and *Bam*HI sites of plasmid pSEM2027 containing a strong synthetic promoter (Fig. 4 top in Bendtsen *et al*.)^64^. The resulting plasmid was cleaved by *Eco*RI and *Bam*HI, and the fragment containing the synthetic promoter and the downstream gene encoding the Venus YFP-RelB fusion was inserted between the *Eco*RI and *Bam*HI sites of plasmid pLG338^65^ (pSEM4045).

Plasmid pSEM4102 carries the *P*_relB_ promoter that transcribes the gene encoding YFP^unstable^, a short lived fluorescent marker (a fusion of the λ CII protein and Venus YFP), degraded by the HflB protease^39^. The plasmid was constructed in two steps. First the region containing the *rrnB*T1T2 terminators and the gene encoding YFP^unstable^ was PCR amplified using primers KpnIT1T2^66^ and VENYDN^39^ and plasmid pSEM3131B^39^ as template. The amplified fragment was digested with *Kpn*I and *Bam*HI and inserted into plasmid pLG338 between the same restriction sites (pSEM3034). The *P*_relB_ promoter region was PCR amplified using primers RelBP_RB1 and RelBP and MG1655 chromosomal DNA as template. The fragment was cut with *Eco*RI and *Bam*HI, and inserted between the *Eco*RI and *Bgl*II sites of pSEM3034, located between the *rrnB*T1T2 terminators and the gene encoding YFP^unstable^. Plasmid pSEM4102m was constructed in a similar way, except that the YFP coding sequence was replaced by the mCherry coding sequence.

The plasmid encoding the ATP sensor QUEEN-7µ^40^ was constructed in two steps. First, the sequence encoding QUEEN-7µ was PCR amplified using the primers promforQ and Q2mdn, and pRSET B QUEEN-7μ^40^ as a template. The PCR product was cut with *Bgl*II and *Hind*III, and inserted between the *Bgl*II and *Hind*III sites of pdCas9-bacteria^67^. In this plasmid (pSEM4151) QUEEN-7μ is produced at sufficient level but the expression level can be further induced by anhydrotetracycline. Next, the QUEEN-7μ expression region (including *tetR*) of pSEM4151 was PCR amplified using primers Tet_AgeI and 7µ_AgeI. The PCR product was digested by *Age*I and inserted at the *Age*I site of pSEM4102m in the orientation that the promoters transcribe QUEEN-7μ downstream of the *rrnB*T1T2 terminators (pSEM4157).

To construct a plasmid for the expression of the hexahistidine-tagged β-lactamase protein, the pEM7/zeo plasmid (Invitrogen) was PCR amplified using the BlaHis1 and BlaHis2. The resulting PCR product was digested with *Acc*65I and circularized by T4 DNA ligase (pSEM3132). DNA sequences of the cloned regions were verified in all plasmids (Eurofins Genomics).

### Purification of β-lactamase

*E. coli* DH5α cells carrying the pSEM3132 plasmid were grown overnight in 150 ml LB containing 100 μg/ml ampicillin. Cells were removed by centrifugation and the cleared supernatant was filtered using a 0.45 μm filter (Millipore). 2 ml of Ni-NTA slurry (Qiagen) was added to the solution, followed by 1 h incubation at 4 °C. A Poly-Prep Chromatography Column (BIO-RAD) was used to collect the Ni-NTA agarose-bound proteins from the mixture. Twenty column volume of washing buffer (50 mM sodium phosphate, pH 7.2, 600 mM NaCl, 10% glycerol) was allowed to flow through the column. β-lactamase was eluted by four column volumes of elution buffer (50 mM sodium phosphate, pH 7.2, 600 mM NaCl, 50% glycerol) containing 250 mM imidazole. The solution was filtered using a 0.2 μm filter (Millipore) and stored at −20 °C. 10 μl of the solution was plated on an LB agar plate containing 100 μg/ml ampicillin, and no colony growth was observed after 10 days of incubation at 37 °C.

### Persister assays

#### Assays performed in the plate reader

Overnight cultures were diluted in supplemented M9 glucose medium and grown to optical density of ~0.1 (600 nm) at 36.6 °C in a temperature controlled microplate reader (FLUOstar® Omega, BMG Labtech) with constant shaking (500 rpm) between the reads. Cultures were treated with ampicillin (150 μg/ml) and the incubation was continued. Samples were taken before the addition of ampicillin, and after 6 and 16 h of ampicillin treatment to determine the number of colony forming units. Purified β-lactamase was added to the cultures before diluting and plating them on LB agar plates. Plates were incubated at 30.0 °C for 48 hours before counting the colonies.

#### Assays performed in culture tubes

Bacteria were diluted 1:100 from dense overnight cultures (in LB or supplemented M9 medium) into 4 × 2 ml of the same medium in conical plastic culture tubes that had been supplemented with ampicillin (100 μg/ml). Culture tubes were agitated at 37 °C. At t = 0 h, t = 1 h, t = 5 h, and t = 24 h one tube per strain was sampled in order to determine the count of colony forming units. Per tube, 1.5 ml of culture was spun down in a microcentrifuge (4 minutes at 13'000 rpm) and the supernatant was removed exactly. Pellets were washed once in sterile PBS, serially diluted in sterile PBS, and plated on LB agar plates. Colony counts were determined after 24 h of incubation at 37 °C.

### Fluorescence Microscopy

Cells were grown at 30 °C for 2 h in LB or for 5 h in supplemented M9 medium containing 30 µg/ml kanamycin before plating them on M9 (glycerol) agarose (1%) pads containing 30 µg/ml kanamycin and supplemented with 0.1 mg/ml amino acids (excluding Phe, Tyr, and Trp to limit background fluorescence). The M9 agarose pad was placed directly on a microscope cover slip and sealed using a custom setup, enabling stable experimental conditions over extended periods of time^53^. We imaged cells using 100X objective every 20 min on a temperature-controlled, automatic microscope (Nikon Eclipse *Ti*). The excitation and emission wavelengths used were 470/515 (YFP^unstable^, 1 s), 580/615 (mCherry, 1 s), 405/520 and 490/520 (QUEEN-7µ, 50 ms and 100 ms, respectively). The images were analyzed using the NIS Elements image analysis software (Nikon), and Fiji^68^. Antibiotic treatment was performed by placing a drop of antibiotic solution on the top of the agarose pad and allowing it to diffuse through it to the other side where the cells were.

### (p)ppGpp measurement

The intracellular level of ppGpp and pppGpp was measured using thin-layer chromatography, as previously described^22^. The measurements were done for SEM3147. The strain was grown in MOPS medium^23^ supplemented with 0.4% glycerol and 17 amino acids 0.1 mg/mL (excluding Phe, Typ, and Trp). The phosphate concentration was 330 μg/mL. The cells were grown exponentially at 30 °C in a water bath for more than 10 generations. At OD_436_ = 0.05, 2 mL were put into ^32^P and in addition, a parallel culture without 32 P was used for OD measurements and used to interpolate the OD used to normalize the final (p)ppGpp measurements. In the culture containing ^32^P, the cells were grown for 2–3 generations, before samples were taken. Four samples were taken within minutes before the temperature shift to 37 °C, where a series of samples were taken from time 0 minutes up to 80 minutes. Each sample was treated as in^22^. In this study, only 1-D PEI Thin layer chromatography was employed. The radioactivity in each spot was determined by the use of a phosphorimager and a medium sample spotted onto a TLC-plate exposed on the same scan allowed a direct measurement of the specific activity of the phosphate.

## Supplementary information

Supplementary tables and figures

## Data Availability

All data generated or analysed during this study are included in this published article (and its Supplementary Information files).
