# Supplementary material for: Birth and Resuscitation of (p)ppGpp Induced Antibiotic Tolerant Persister Cells
Source: Sci Rep. 2019 Apr 15;9:6056. doi: 10.1038/s41598-019-42403-7 (PMC6465370; doi:10.1038/s41598-019-42403-7)
Supplement: Supplementary file 1 — Supplementary tables and figures [file 41598_2019_42403_MOESM1_ESM.pdf]

Supplementary information

Birth and Resuscitation of (p)ppGpp Induced Antibiotic Tolerant Persister Cells

Mikkel Skjoldan Svenningsen[1], Alexandra Veress[2], Alexander Harms[2], Namiko Mitarai[1*], and Szabolcs Semsey[2]*

[1]Niels Bohr Institute, University of Copenhagen, Blegdamsvej 17, 2100 København Ø, Denmark.

[2]Centre for Bacterial Stress Response and Persistence, Department of Biology, University of Copenhagen, Ole Maaløesvej 5, 2200 København N, Denmark.

*Correspondence: mitarai@nbi.ku.dk, semseys@yahoo.com;

Supplementary Table

Table S1. Strains, plasmids and oligonucleotides.

**STRAINS**		
**Name**	**Features**	**Reference**
JF858	*valS*^{ts}	Reeh et al[1]
E.coli K12 MG1655	F$^-$ λ$^-$ *ilvG*$^-$ *rfb*-50 *rph*-1	Our lab collection
SEM3147	MG1655 *valS*^{ts}	This work
EM477	MG1655 *rpoS::mCherry*	Maisonneuve et al[2]
EM477VTS	MG1655 *valS*^{ts} *rpoS::mCherry*	This work
SEM3156	MG1655Δ*relA::cm*	This work
SEM3157	MG1655 *valS*^{ts}Δ*relA::cm*	This work
AHK205	MG1655Δ*sulA*::FRTΔ*lon rpoS::mCherry*	This work
SEM4178	AHK205*valS*^{ts}	This work
AHK250	MG1655Δ10TA	Harms et al[3]
SEM4202	AHK250*valS*^{ts}	This work

AHK034	MG1655Δ*relA* Δ*spoT*	Harms et al[3]
AHK298	AHK034 *rpoB(Y396D)*	This work
AHK302	AHK034 *rpoC*Δ*(T212-Q229)*	This work
AHK303	AHK034 *rpoC(L127-L132)*$_2$	This work

PLASMIDS

**Name**	**Features**	**Reference**
pRFB122	cm^R cassette	Fekete & Chattoraj[4]
pCP20	FLP recombinase	Datsenko & Wanner[5]
pSEM3131B	Venus YFP	Mitarai et al[6]
pLG338	Low copy plasmid	Stoker et al[7]
pSEM4045	Venus YFP-RelB fusion in pLG338	This work
pSEM4102	P_{relB} promoter- *yfp*^{unstable} fusion	This work
pSEM4102m	P_{relB} promoter- *mcherry*^{unstable} fusion	This work
pRSET B QUEEN-7μ	QUEEN-7μ	Yaginuma et al[8]
pSEM4157	QUEEN-7μ and P_{relB} promoter- *mcherry*^{unstable} fusion	This work
pSEM3132	hexahistidine tagged β-lactamase	This work

PRIMERS

**Name**	**Sequence (5′ → 3′)**
ValS1	AGTCGAAAACCGCGAATCGAAAGGTTC
ValS_Nco	TTGGCCATGGTTTAGTTCCTCACCTTGTCGTTACAGCGCGGCGATAACAGCCTGCTGT
ValS2	CAGGACTGGTGTATCTCTCGTCAGTTGT
ValSREC	CCATTTCTGTAAGAGATAAAAAAGGCCGGAGCATGCTCCGGCCTTCGTTTTCATCACTGTGTTTTGA CAG CTGGCGAAAGGGGGATGTGCTGC
RelAupCm	TACTTTTCTCGCGCGTTAAATAGTTGCGATTTGCCGATTTCGGCAGGTCTGGTCCCTAAAGGAGAGG ACGTGCCGTTACGCACCACCCCGTC
RelAdnCm	GCTATCATATGTAGATACAGTATATATCAATCTACATTGTAGATACGAGCAAATTTCGGCAATTACG CCCCGCCCTGCCACTC
RelYdn	AAAAGGATCCTTATCAGAGTTCATCCAGCGTCACAC
RelYJ2	CGCAGGTACCGG TAGCATTAACCTGCGTATT
RelYup	AAAACTGCAGAGGAGGCCCTTATGGCAACTAGCGGCATGGT
RelYJ1	TACCGGTACCTGCGGTTCCAGAACCTTTGTATAGT

promforQ	AAAACTGCAGATCTAGATTCACAAACCTTTCGCGGTATGG TATAATTTTGTTTAACTTTAAGAAGG
Q2mdn	CGATCAAGCTTCGAATTCACTTCATTTCCGC
Tet_AgeI	TTTTACCGGTGACGTCTTAAGACCCACTTTCAC
7µ_AgeI	TTTTACCGGTTCGAATTCACTTCATTTCCGC
BlaHis1	CGTAAGGTACCTGTCAGACCAAGTTTACTCATATATAC
BlaHis2	TGACAGGTACCTTACGAGGAGTGGTGGTGGTGGTGGGAGGAGCCCCAATGCTTAATCAGTGAGG CACCTATC
RelBP_RB1	AAAAGAATTCTCTCTAGATCTCACAAAGCGGACA GTGATCAC
RelBP	AAAAGGATCCGTCTTACACCTCTTGTAATT

Supplementary Figures

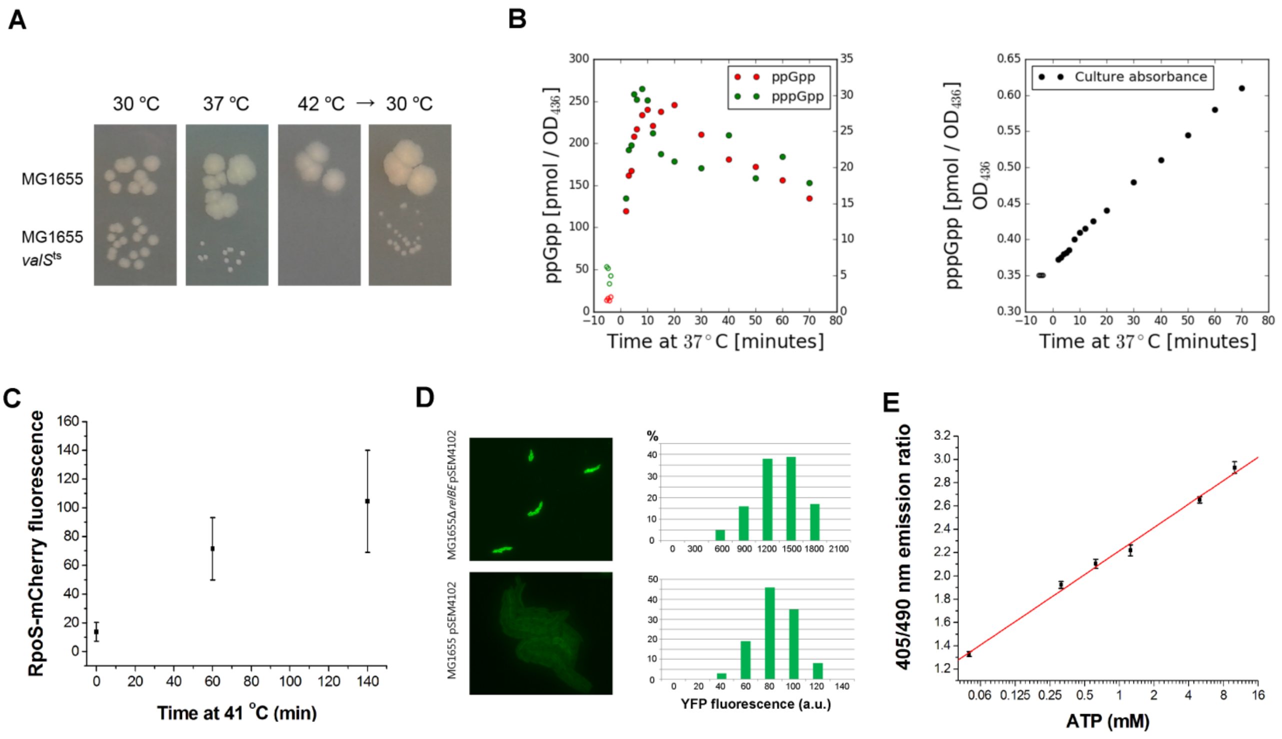

Figure S1. (A) Temperature-sensitive growth and reversible growth inhibition of MG1655 *valS*^{ts} cells (SEM3147). Cells were grown overnight on LB agar plates at the indicated temperatures. The growth of MG1655 *valS*^{ts} cells was strongly inhibited at 42°C (3rd panel) but further incubation at 30°C allowed cell resuscitation and development of colonies (4th panel). (B) The intracellular level of (p)ppGpp during a temperature shift from 30 to 37 °C (left panel). Both guanosine tetra- (ppGpp) and pentaphosphate (pppGpp) are strongly elevated at 37 °C, approximately 16-fold (tetra) and 6-fold (penta) respectively, compared to the level at 30 °C. At 80 minutes, the levels are still approximately 9-fold (tetra) and 3-fold (penta). The concentration of (p)ppGpp was measured using thin-layer chromatography. The growth curve of the culture during sampling for (p)ppGpp measurements is shown on the right panel. The culture is still growing after the shift to 37 °C but at a lower rate. The growth medium was MOPS glycerol 0.4 % with 17 amino acids 0.1 mg/ml.

(C) Limitation of ValS activity in MG1655 *valS*^{ts} cells carrying a chromosomal *rpoS::mcherry* gene fusion results in increased RpoS-mCherry levels, indicating elevated levels of (p)ppGpp. Cells were grown at 30 °C until mid-log phase before incubation at 41 °C and fluorescence microscopy. RpoS-mCherry fluorescence was measured in single cells and average values were plotted as a function of time spent at 41 °C. (D) Expression of the YFP^{unstable} reporter from the P_{relB} promoter in MG1655 (bottom left) and MG1655*ΔrelBE* cells (top left) carrying the pSEM4102 plasmid P_{relB}::*yfp*^{unstable}). Distributions of fluorescence levels in single cells are shown on the right. (E) The ATP sensor QUEEN-7μ was purified as described previously[8] and the purified protein was diluted in supplemented M9 medium to 0.8 mg/ml concentration. The intensity of the emitted light at 520 nm upon excitation at 405 and 490 nm was determined at different ATP concentrations in the microscope at 37 °C. The ratio of the light intensities (405/490 nm) was calculated from 5 measurements. ATP batches used for the calibration were sourced from Sigma-Aldrich, Invitrogen, and Promega. Data was fitted to the equation $[ATP] = 2^{(ratio-2.214)/0.2015}$ (R^2=0.998).

A

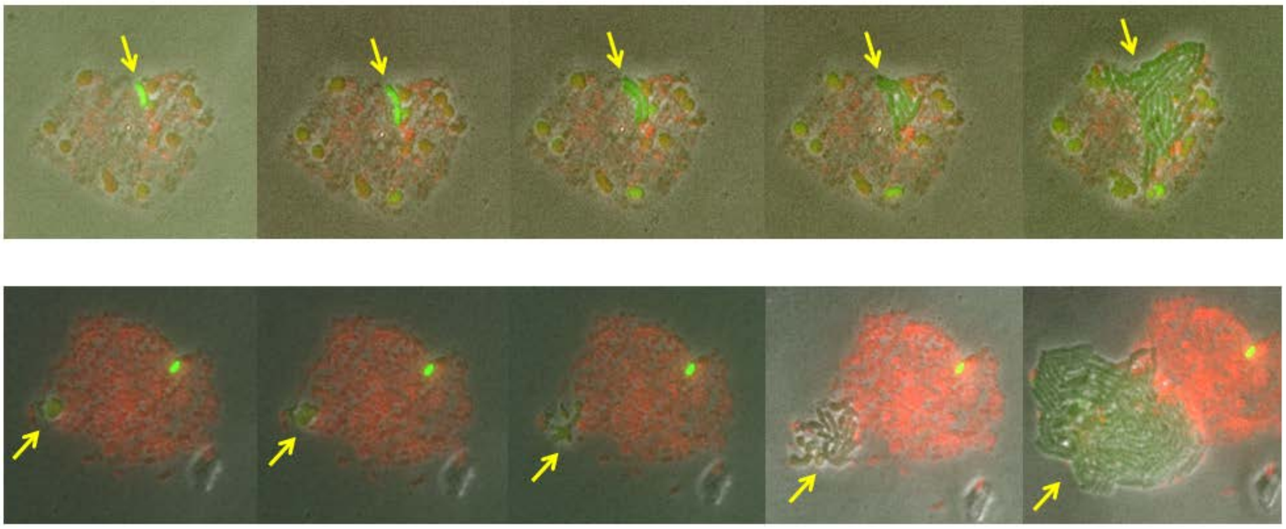

B

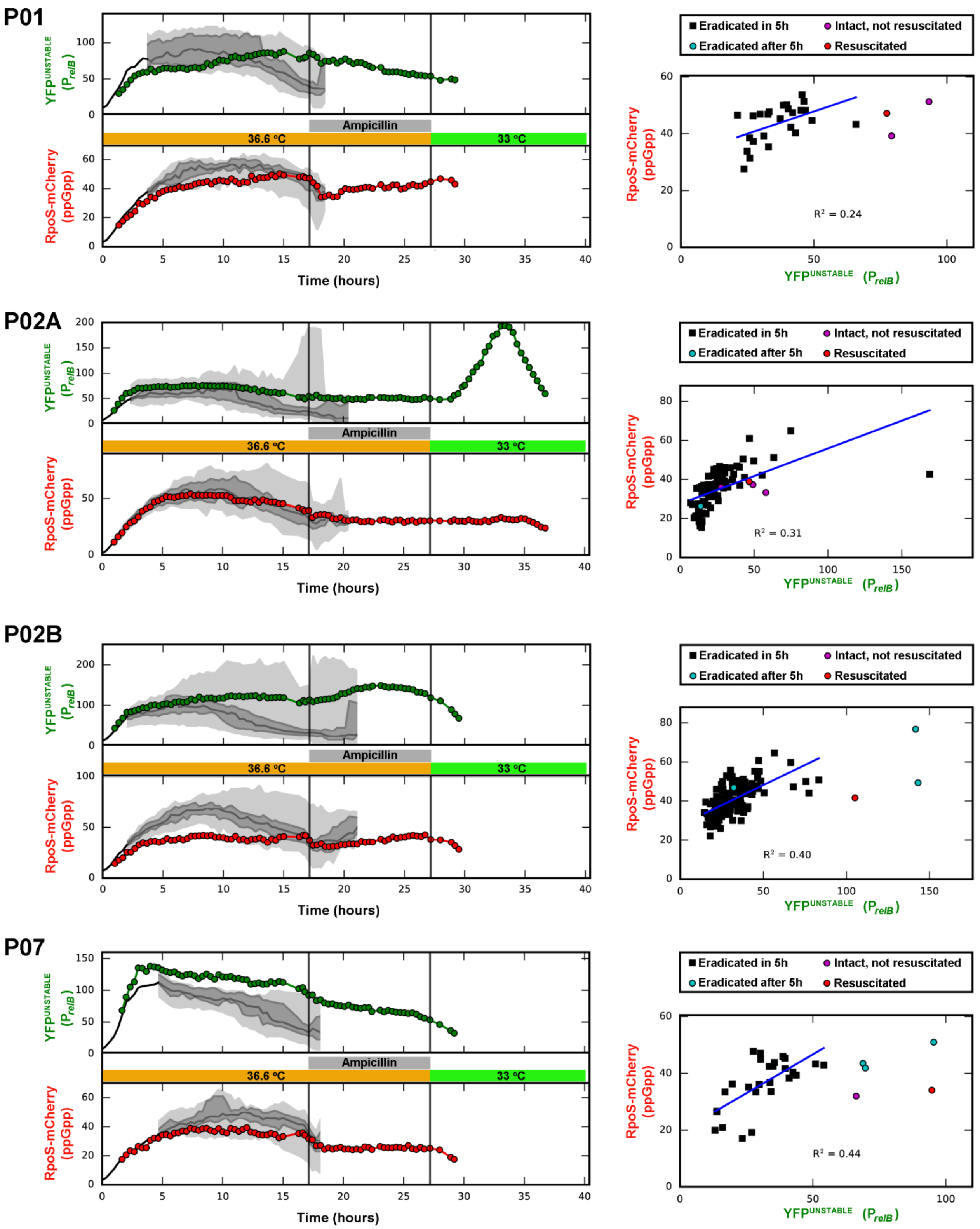
P01
P02A
P02B
P07
YFP^UNSTABLE (PrelB)
RpoS-mCherry (ppGpp)
Ampicillin
36.6 ºC
33 ºC
Time (hours)
Eradicated in 5h
Intact, not resuscitated
Eradicated after 5h
Resuscitated
R² = 0.24
R² = 0.31
R² = 0.40
R² = 0.44

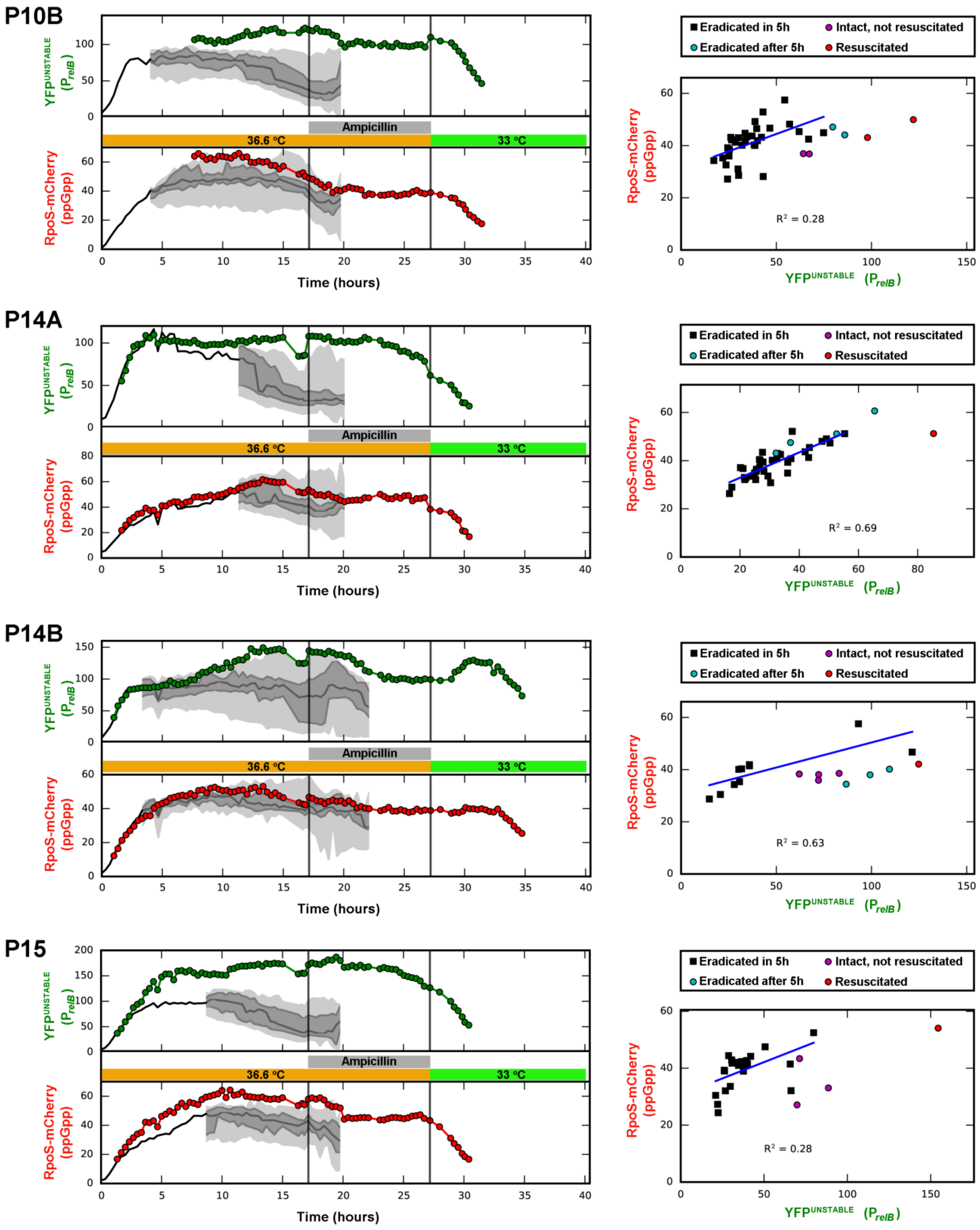
P10B
P14A
P14B
P15
YFP^UNSTABLE (PrelB)
RpoS-mCherry (ppGpp)
Ampicillin
36.6 °C
33 °C
Time (hours)
Eradicated in 5h
Intact, not resuscitated
Eradicated after 5h
Resuscitated
R² = 0.28
R² = 0.69
R² = 0.63
R² = 0.28

C

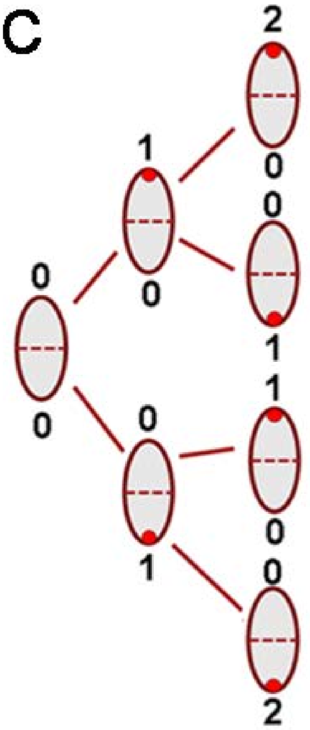

Persister cell	Age of the persister's older pole	Sister's fate (cell age)
P01	2	Eradicated (16h)
P02A	2	Intact but not resuscitated (37h)
P02B	3	Divided (1h)
P07	1	Eradicated (22h)
P10A	1	Eradicated (24h)
P10B	1	Eradicated (13h)
P11	1	Intact but not resuscitated (37h)
P14A	1	Divided (5h)
P14B	1	Divided (4h)
P15	1	Divided (7h)

Figure S2. (A) Resuscitation of ampicillin survivors after β-lactamase treatment in the experiment presented in Figure 2. The top panel shows the regrowth of a normal cell with a high level of the *relB* promoter reporter. The bottom panel shows the resuscitation of an L-form-like cell. (B) Supplementary data to Figure 2. Time-series of YFP^{unstable} (green symbols) and RpoS-mCherry (red symbols) levels and corresponding pre-ampicillin distributions for 8 additional colonies. (C) Asymmetry in the fate of the persister and its sister. Persister cells correspond to cells observed in Figure 2A, 2B (referred as P10A and P11, respectively) and Figure S2. The age of the older pole of persister cells at birth is indicated in the middle column and the fates of the sister cells are shown on the right.

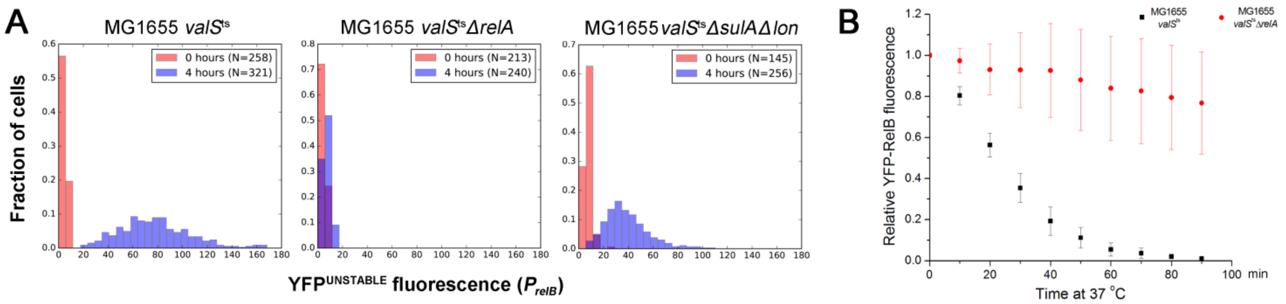

Figure S3. (A) Changes in the distribution of the *relB* promoter reporter (YFP^{unstable}) upon limitation of tRNA charging in MG1655*valS*^{ts}, MG1655*valS*^{ts}Δ*sulA*Δ*lon*, and MG1655 *valS*^{ts}Δ*relA* cells carrying the plasmid borne *relB* promoter-*yfp*^{unstable} fusion (pSEM4102). Cells were incubated in a temperature controlled microscope at permissive temperature (32 °C) in the presence of 0.15 mg/ml SHX. Images were taken at 0 time and after 4 hours. (B) Stability of the YFP-RelB fusion upon limitation of tRNA charging. MG1655 *valS*^{ts} and MG1655 *valS*^{ts}Δ*relA* cells carrying a plasmid borne YFP-RelB reporter with a synthetic promoter were grown at 30 °C for 2h before placing them in the microscopy chamber at semi-permissive temperature (36.6 °C). The development of YFP fluorescence was recorded for single cells. Although there is some residual reporter production at the semi-permissive temperature in this experimental setup, the decay rate to the lower steady state level reflects the protein half-life. The half-life of YFP-RelB can be used as a reliable proxy of the stability of RelB, because GFP and its derivatives co-degrade with their fusion partner[9,10]. In agreement with the activation of the *relB* promoter (Figure 3), the RelB protein was quickly degraded in the MG1655 *valS*^{ts} cells but remained rather stable in the MG1655 *valS*^{ts}Δ*relA* cells (Figure S3). The ~20 min half-life of the RelB protein observed after (p)ppGpp induction by inhibition of tRNA charging agrees well with the ~17 min half-life reported for the RelB protein when (p)ppGpp synthesis was induced by SHX[2].

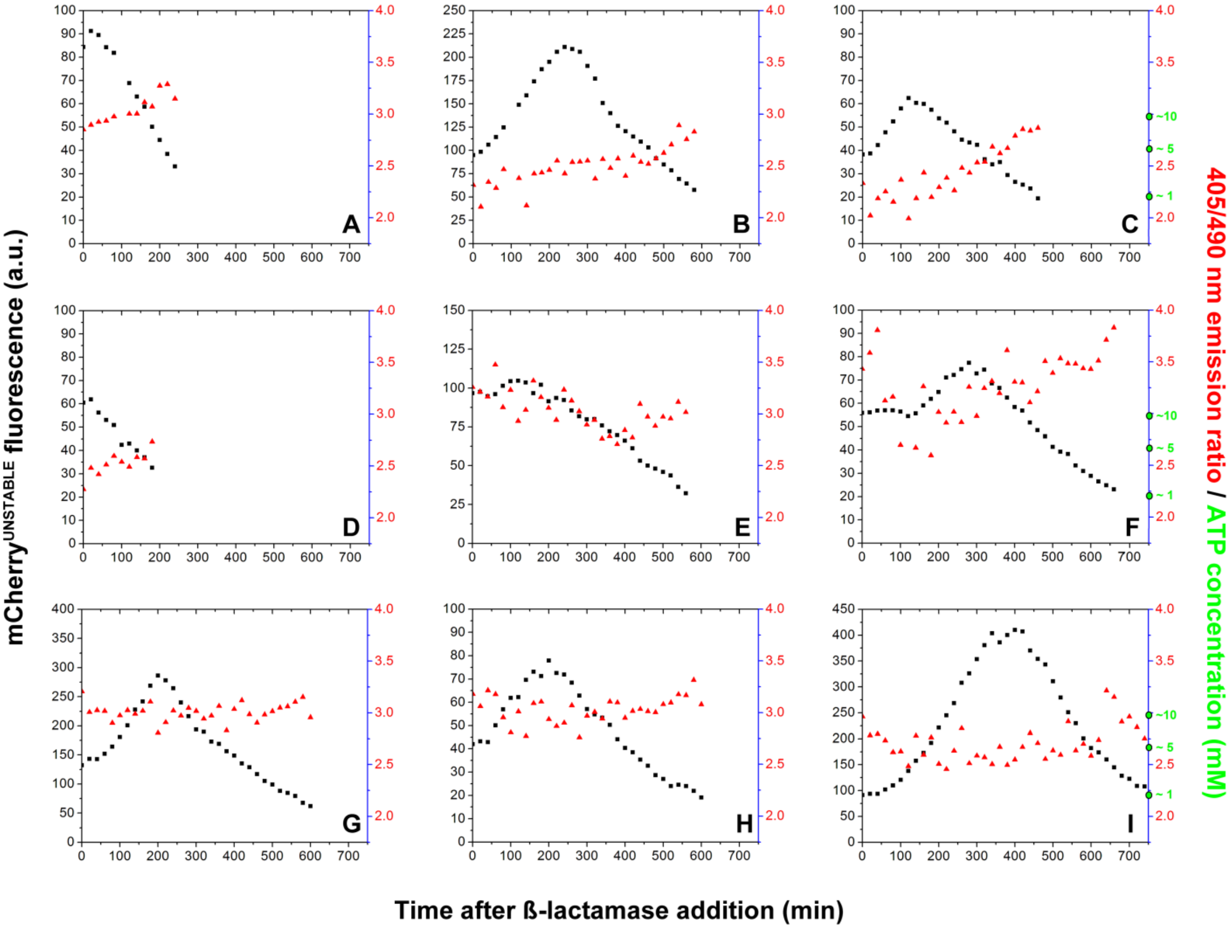

Figure S4. Supplementary data to Figure 4. Development of ATP levels determined using ATP sensor QUEEN-7µ[8] (red triangles, 405/490 nm emission ratio) and mCherry^{unstable} levels (black squares) during resuscitation of persisters are shown. β-lactamase was added to remove ampicillin at time 0. Fluorescence levels were recorded every 20 minutes. The last time point represents cell division and values shown are means of the fluorescence intensities observed in the two daughter cells. See also Figure S1D for validation of the ATP sensor QUEEN-7µ in our experimental system.

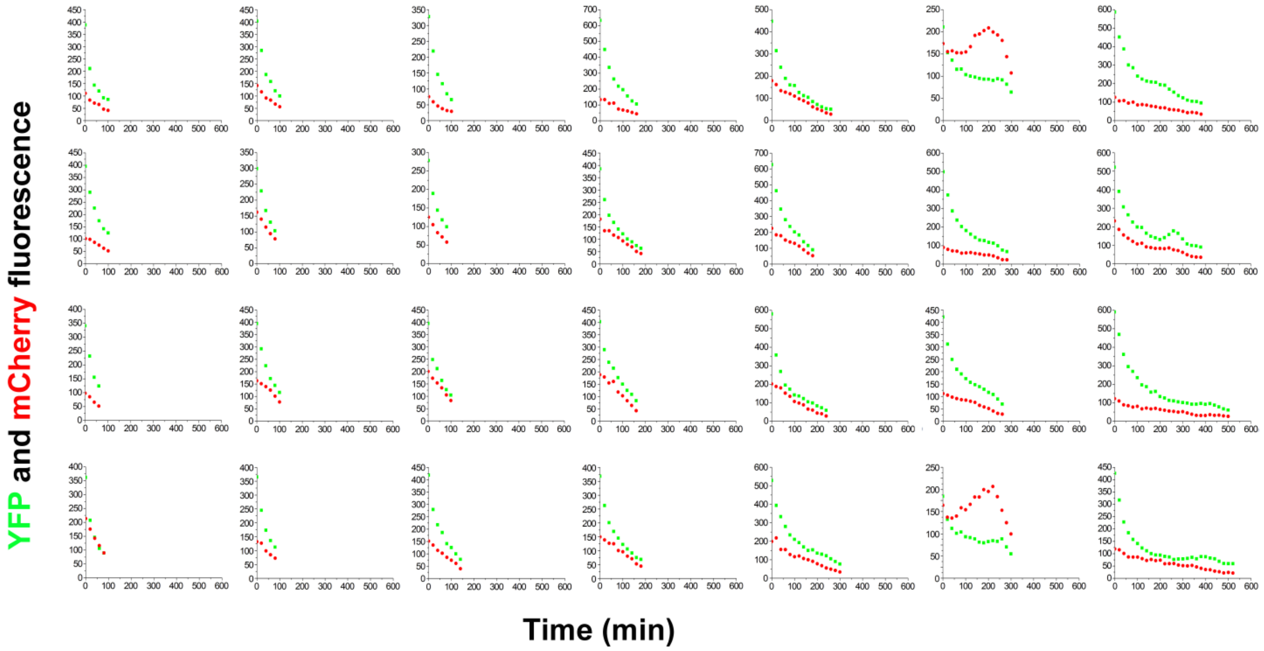

Figure S5. Quantification of the development of mCherry and YFP fluorescence in 28 persister cells obtained in the experiment shown in Figure 5. The last time point represents cell division and values shown are means of the fluorescence intensities observed in the two daughter cells.

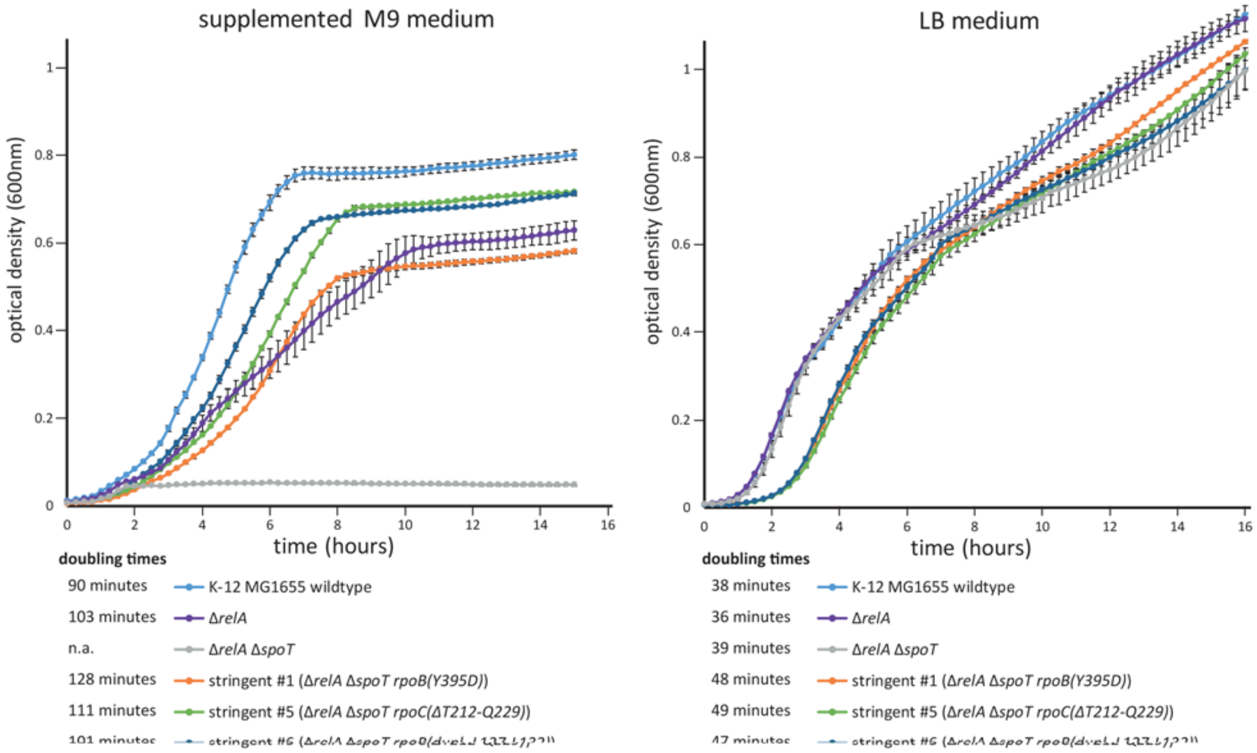

Figure S6. Dense overnight cultures of bacteria were diluted 1:500 from LB or supplemented M9 medium into 225 µl of the same medium in wells of sterile 96 well microtiter plates. All wells were overlayed with 50 µl of mineral oil and continuously agitated in a microtiter plate reader at 37 °C. The optical density of bacterial cultures and blank wells with sterile medium were recorded over time and the measurements of blank wells were subtracted from the values obtained with bacterial cultures at the same time point. Data points and error bars represent average and standard deviation of three independent wells of a representative experiment.

Supplementary References

1 Reeh, S., Pedersen, S. & Friesen, J. D. Biosynthetic regulation of individual proteins in relA+ and relA strains of Escherichia coli during amino acid starvation. *Mol Gen Genet* **149**, 279-289, (1976).

2 Maisonneuve, E., Castro-Camargo, M. & Gerdes, K. (p)ppGpp controls bacterial persistence by stochastic induction of toxin-antitoxin activity. *Cell* **154**, 1140-1150, (2013).

3 Harms, A., Fino, C., Sorensen, M. A., Semsey, S. & Gerdes, K. Prophages and Growth Dynamics Confound Experimental Results with Antibiotic-Tolerant Persister Cells. *MBio* **8**, (2017).

4 Fekete, R. A. & Chattoraj, D. K. A cis-acting sequence involved in chromosome segregation in Escherichia coli. *Mol Microbiol* **55**, 175-183, (2005).

5 Datsenko, K. A. & Wanner, B. L. One-step inactivation of chromosomal genes in Escherichia coli K-12 using PCR products. *Proc Natl Acad Sci U S A* **97**, 6640-6645, (2000).

6 Mitarai, N., Jensen, M. H. & Semsey, S. Coupled positive and negative feedbacks produce diverse gene expression patterns in colonies. *MBio* **6**, (2015).

7 Stoker, N. G., Fairweather, N. F. & Spratt, B. G. Versatile low-copy-number plasmid vectors for cloning in Escherichia coli. *Gene* **18**, 335-341, (1982).

8 Yaginuma, H. *et al.* Diversity in ATP concentrations in a single bacterial cell population revealed by quantitative single-cell imaging. *Sci Rep* **4**, 6522, (2014).

9 Cameron, D. E. & Collins, J. J. Tunable protein degradation in bacteria. *Nat Biotechnol* **32**, 1276-1281, (2014).

10 Shah, I. M. & Wolf, R. E., Jr. Sequence requirements for Lon-dependent degradation of the Escherichia coli transcription activator SoxS: identification of the SoxS residues critical to proteolysis and specific inhibition of in vitro degradation by a peptide comprised of the N-terminal 21 amino acid residues. *J Mol Biol* **357**, 718-731, (2006).
